# Original investigation: evolution of long-term cardiac tumours in patients with tuberous sclerosis

**DOI:** 10.1186/s13023-026-04302-9

**Published:** 2026-05-07

**Authors:** Nathalia Conci Santorio, Anna Christina de Lima Ribeiro, Nilson Bossle Conci, Gardênia da Silva Lobo Oishi, Pandreli Testa Santorio, Maria Rosa Quadrado Matos, Nana Miura Ikari, Fábio Fernandes, Viviane Tiemi Hotta

**Affiliations:** 1 Heart Institute (InCor), Hospital das Clínicas HCFMUSP, Faculdade de Medicina, Universidade de São Paulo, São Paulo, SP Brazil; 2 Unimed Sul-Capixaba, Cachoeiro de Itapemirim, ES Brazil; 3 Fleury Medicina e Saúde, São Paulo, SP Brazil; 4 Dr. Enéas Carvalho de Aguiar Avenue, 44 - Cerqueira César, São Paulo, 05403-900 SP Brazil

**Keywords:** Tuberous sclerosis complex, Cardiac rhabdomyoma, Arrhythmia

## Abstract

**Background:**

The tuberous sclerosis complex is an autosomal dominant genetic disorder caused by mutations in the TSC 1 or 2 genes. Cardiac rhabdomyomas are the most frequent initial manifestation and leading cause of mortality in children under 10 years of age. Data on Brazilian patients with rhabdomyomas are scarce.

**Objectives:**

This study aims to describe the diagnostic aspects and clinical features observed during the follow-up at a high-complexity cardiology centre.

**Methods:**

This was a retrospective, descriptive, single-centre study, based on medical records. Patients of all age groups and sexes were included, with a confirmed diagnosis of tuberous sclerosis and at least two serial transthoracic echocardiograms performed at the service from January 1997 to January 2024. Patients with uncertain diagnoses and incomplete records were excluded.

**Results:**

Among the 69 patients evaluated, 42 (60.86%) had cardiac tumours, with 41 rhabdomyomas and one pericardial lipoma, with a mean follow-up time of 6 years. The median age of the cohort at first evaluation was 3.5 years [1.0; 15.8]. Multiple tumours were observed in 75.6% of cases. Most patients with rhabdomyomas were asymptomatic at both evaluations (73.8% and 85.71%, respectively); however, episodes of arrhythmia were recorded in 21.43% of the sample during follow-up. Only one patient presented with ventricular dysfunction, and one patient required surgical treatment, resulting in death. Incomplete involution of the mass occurred in 76.2% of cases, complete regression in 16.7%, and maintenance, increase, or need for surgical treatment in 7.2%.

**Conclusions:**

Our data indicate a considerable prevalence of arrhythmias and the persistence of identifiable masses throughout follow-up in a Brazilian cohort of patients with tuberous sclerosis complex, emphasizing the clinical relevance of persistent lesions as potential arrhythmogenic substrates requiring long‑term surveillance.

**Supplementary Information:**

The online version contains supplementary material available at 10.1186/s13023-026-04302-9.

## Introduction

The tuberous sclerosis complex (TSC) is a rare autosomal dominant genetic disorder, with an incidence of 1 in 6,760 to 1 in 13,520 live births, showing no sex predilection [1]. It results from mutations in the TSC1 or TSC2 genes, which encode the regulatory proteins hamartin and tuberin, respectively [2].

Neurological and cutaneous manifestations are most common. Epilepsy, present in 80–90% of patients, often begins in the first year of life [3]. Cortical tubers, which are responsible for the disease’s name, occur in up to 90% of cases and serve as potential substrates for epileptic seizures [4]. Hypomelanotic lesions, usually present at birth, also affect 90% of patients [5].

Renal manifestations are the leading cause of morbidity and mortality in adults with TSC [6]. Renal angiomyolipomas are present in 48% of patients [7] and can lead to hypertension, progressive renal function impairment and an increased risk of hemorrhagic complications [8].

On the other hand, in patients under 10 years of age, cardiac rhabdomyomas are the most frequent cause of death [9]. They are found in 34.3% [10] to 48% [11] of cases, and it is estimated that 51% to 86% of rhabdomyomas are associated with TSC [12].

These tumours tend to develop between the 20th and 30th weeks of gestation, and are influenced by maternal hormones [9]. They are more common in patients with TSC2 mutations, which may be associated with more severe disease manifestations [11].

Rhabdomyomas vary significantly in size and are multiple in 90% of cases, typically located in the right and left ventricles with similar distribution [9]. Most regress spontaneously during the first year of life and are observed less frequently after age two [11].

**Fig. 1:**
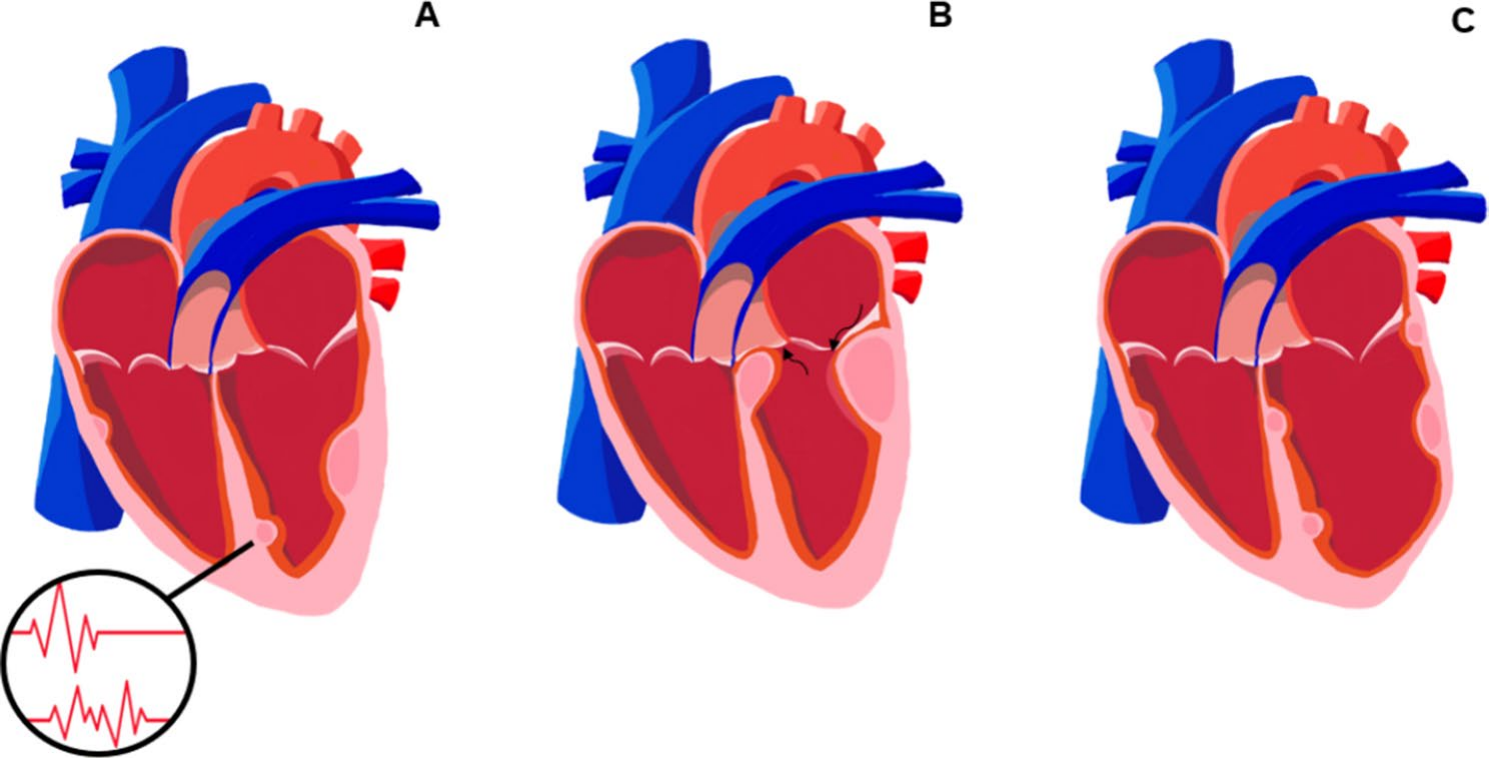
Main manifestations associated with cardiac rhabdomyomas. Representative illustration of the main manifestations associated with cardiac rhabdomyomas. **A**: Tumour masses as substrates for arrhythmias. **B**: Obstruction of the inflow and/or outflow tracts of the ventricles with haemodynamic repercussions. **C**: Heart failure associated with extensive tumour infiltration. Source: Santorio NC (2025)

In most cases, they are asymptomatic, but they may cause clinical repercussions soon after birth or within the first year of life [9]. This can occur due to obstruction of the inflow or outflow tracts of the ventricles, leading to significant pressure gradients, myocardial infiltration causing heart failure, or acting as substrates for complex arrhythmias [9], as shown in the Fig. 1. In some cases, large tumours can result in significant haemodynamic compromise and even intrauterine death [13].

Transthoracic echocardiography (TTE) is the preferred imaging modality for diagnosis and follow-up due to its accessibility, repeatability, and haemodynamic assessment. Echocardiography is recommended for fetuses when abnormalities compatible with heart disease are observed on morphological ultrasound, for all paediatric patients under three years of age, and for adults with clinical manifestations [14]. For asymptomatic patients with cardiac involvement, TTE is advised every 1–3 years until tumour regression [14].

Cardiac magnetic resonance imaging (CMR) is the gold standard for characterizing cardiac masses and planning surgery in atypical or complex cases [15, 16].

Surgical resection is considered for tumours that cause severe haemodynamic impact or refractory arrhythmias [9]. Mammalian target of rapamycin (mTOR) inhibitors may be used in symptomatic patients to delay or avoid surgery [17].

The TSC diagnostic criteria, first established in 2012 [18] and updated in 2021 [14], combine clinical and genetic findings. Despite advancements in diagnostic criteria, diagnosis remains challenging in Brazil owing to limited access to genetic tests, imaging, and specialized care.

In 2017, Rosset et al. published the first Brazilian genotype-phenotype study, identifying mutations in 89% of 53 patients and rhabdomyomas in 33% of the sample. The overall prevalence of TSC2 mutations was 2.5 times higher than that of TSC1 mutations, with no significant differences in the prevalence of cardiac tumours [19].

In 2024, Camargo et al. published data on 63 fetuses with rhabdomyomas and reported a 17.4% mortality rate, with 72.7% of deaths occurring prenatally or early postnatally [20].

This study aimed to evaluate the clinical outcomes of patients with cardiac tumours and to conduct an evolutionary assessment of cardiac tumours through serial echocardiography.

## Methods

This retrospective, descriptive, single-centre study included paediatric (0 to 18 years) and adult (over 18 years) patients of both sexes with a confirmed diagnosis of TSC according to the updated 2021 criteria [14], covering the period from January 1997 to January 2024. Patients were selected from the outpatient clinics of Cardiomyopathies, Pulmonology, and Congenital Heart Disease Units of the Heart Institute, Faculty of Medicine, University of São Paulo.

An initial electronic search using the international disease classification was performed in the institution’s electronic medical record system. The data were independently reviewed by two researchers. Patients with at least two serial TTEs performed at the institution were included, while those with uncertain diagnoses or incomplete records were excluded.

Given the study’s characteristics, individual informed consent was waived, with confidentiality and data protection ensured. This investigation was conducted in accordance with the principles outlined in the Declaration of Helsinki. The study was approved by the Medical Ethics Committee of the Hospital das Clínicas, Faculty of Medicine, University of São Paulo.

Anthropometric data, sex, ethnicity, and age at diagnosis were collected. Body surface area was calculated using the Haycock formula [21] for patients under 18 years of age and the Dubois formula [22] for adults.

Systemic manifestations (neurological, renal, pulmonary, dermatological, ophthalmological), malignant neoplasms, the use of mTOR inhibitors, and other disease-associated features were described.

In patients with cardiac tumours, symptoms (dyspnea, chest pain, syncope, tachycardia/palpitations) and cardiologic exams (TTE, Holter, CMR) were evaluated. The first and last TTEs were reviewed according to reference values and guidelines from the American Society of Echocardiography [23]. The data included cardiac chamber dimensions, left ventricular ejection fraction (LVEF) assessed by the Teichholz method [24] and the presence of pericardial effusion. Right ventricular dimensions were assessed qualitatively due to heterogeneity in imaging windows between paediatric and adult exams.

For patients who began follow-up between 0 and 18 years of age, the Z-score for echocardiographic measurements was calculated according to Lopez et al. [25]. The left atrium diameter was assessed according to the scoring system proposed by Pettersen et al. [26], as it was not covered by the previous reference. For individuals who exceeded 18 years of age during follow‑up, Z‑score analysis was maintained to preserve longitudinal comparability, despite lack of formal validation in adults.

Rhabdomyomas were characterized by number (single/multiple), location (ventricles/atria), and haemodynamic impact (presence of flow obstruction signs).

Serial TTEs were used to classify tumour evolution: complete/incomplete regression, growth, or recurrence. The outcomes included surgical intervention, heart transplant, and all-cause mortality.

Holter abnormalities were described if the arrhythmia burden exceeded 1%. Ventricular arrhythmias were classified as simple or complex, with the latter defined by the presence of sustained ventricular tachycardia, nonsustained ventricular tachycardia, or ventricular fibrillation. CMR was used to assess myocardial fibrosis via the late enhancement technique. Genetic mutation data were recorded when available.

Patient mortality was cross-referenced using the national taxpayer database, enabling mortality assessment in patients with and without cardiac involvement.

### Statistical analysis

Normality of quantitative variables was assessed using the Kolmogorov–Smirnov test. For normally distributed variables, descriptive statistics are presented as means and standard deviations (means ± SDs), whereas non-normally distributed variables are reported as medians and interquartile ranges (IQRs, 25th–75th percentiles). Qualitative variables are expressed as absolute (n) and relative (%) frequencies.

Associations between unpaired categorical variables were tested using Fisher’s exact or Chi-square tests. Paired categorical comparisons were performed using McNemar’s or Stuart-Maxwell tests.

Comparisons of unpaired quantitative data were performed via the Student’s t-test for normally distributed variables or the Wilcoxon-Mann-Whitney test for non-normally distributed variables. Age comparisons across three groups were performed via the Kruskal-Wallis test. Paired quantitative data were analyzed using paired t-tests or Wilcoxon paired tests.

An analysis was performed to evaluate the associations between clinical progression and certain individual variables. Additionally, a multivariate analysis using ordinal proportional odds logistic regression [27] was conducted to identify potential factors associated with unfavorable rhabdomyoma progression.

No relevant missing data were present for the variables included in the main comparative or regression analyses. Patients with uncertain diagnoses or incomplete medical records were excluded during the selection phase. No imputation methods were applied.

A significance level of 5% was adopted for all the statistical tests, which were considered two-tailed. All the statistical calculations were performed via R software version 4.2.2 (R Core Team, 2022) by an independent statistical service.

## Results

Initially, 89 records of TSC-diagnosed patients were included. Of these, 20 records were excluded due to incomplete data or uncertain diagnosis. As a national reference, the institution received referrals from multiple geographic regions across Brazil. No systematic international referrals occurred during the study period.

**Fig. 2:**
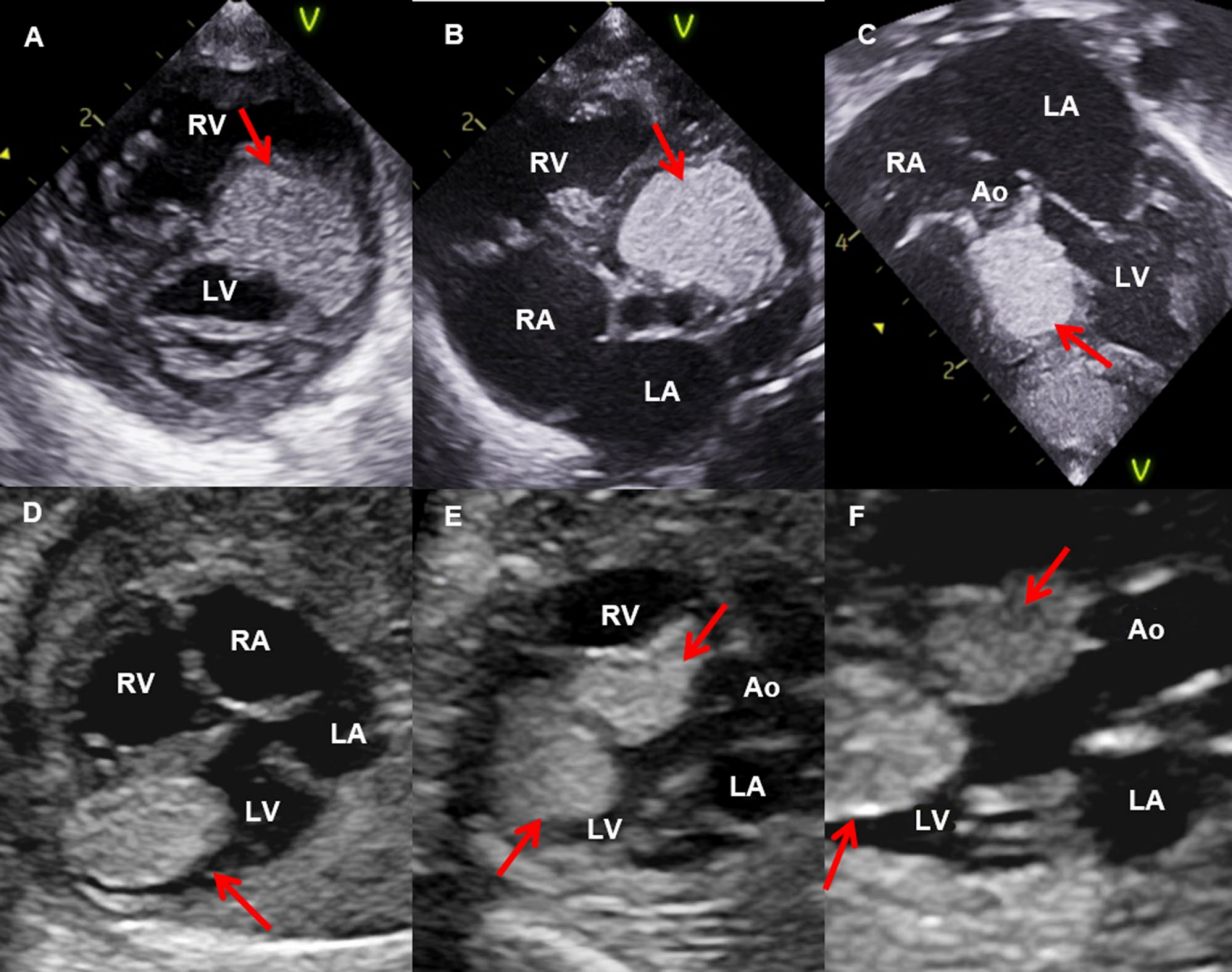
Cardiac rhabdomyomas observed by transthoracic and foetal echocardiography. In **A**, **B**, and **C**, transthoracic echocardiography images show an echogenic mass (arrows) adhered to the interventricular septum, invading and causing obstruction of the left ventricular outflow tract. These findings are demonstrated in the parasternal short-axis view of the ventricles (**A**), short-axis view of the aortic valve (**B**), and apical five-chamber view (**C**). In **D**, **E**, and **F**, foetal echocardiography images reveal echogenic masses (arrows) attached to the interventricular septum and the apical region of the left ventricle. These are shown in the four-chamber view (**D**) and five-chamber views (**E** and **F**). No obstruction of the left ventricular inflow or outflow tracts was observed, with preserved aortic valve opening (**F**). RA: right atrium; LA: left atrium; RV: right ventricle; LV: left ventricle; Ao: aorta. Exams performed at the Paediatric Echocardiography Department of the Heart Institute

**Fig. 3:**
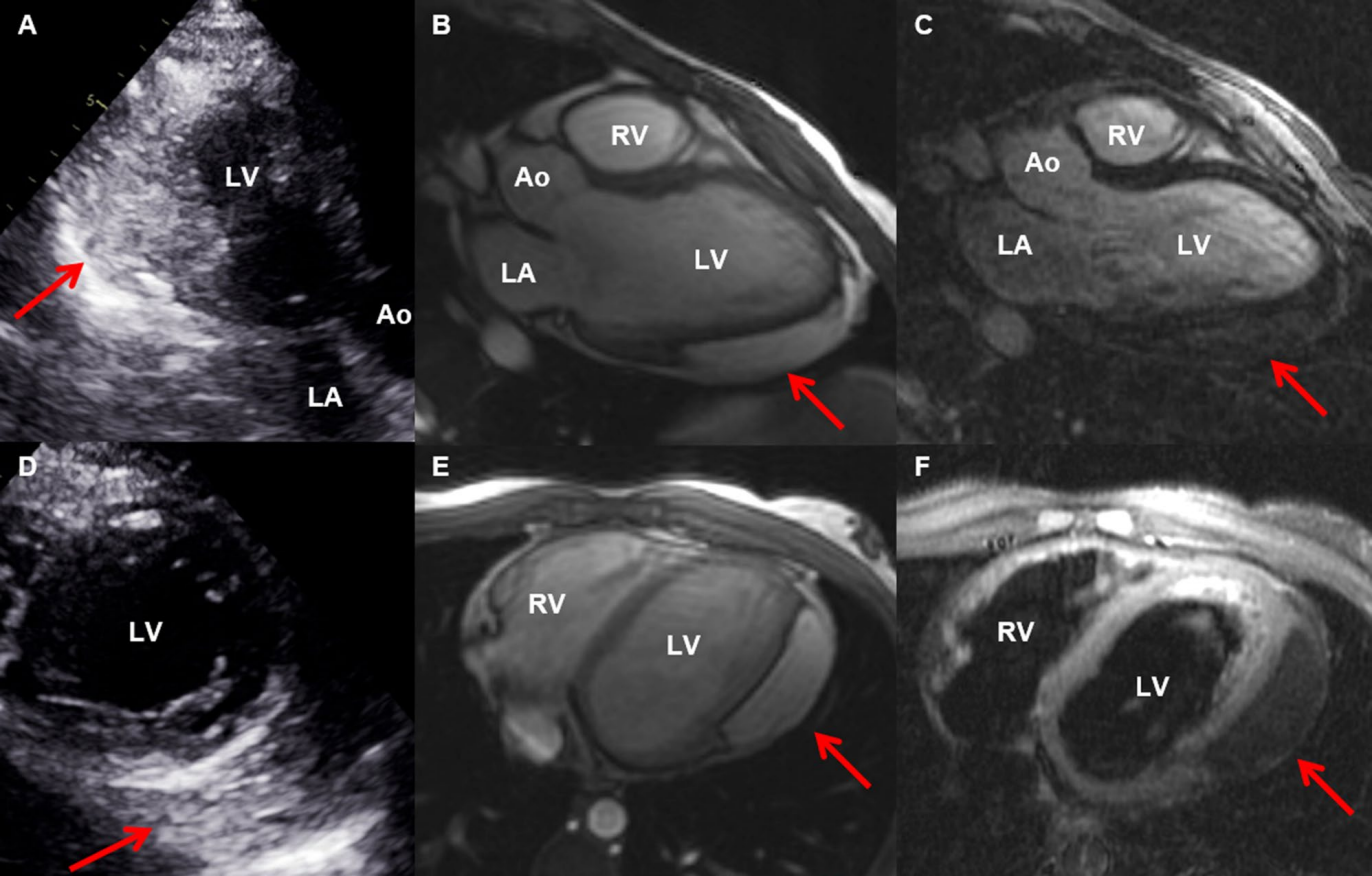
Pericardial lipoma observed via transthoracic echocardiography and cardiac magnetic resonance imaging. Transthoracic echocardiography (**A** and **D**) and cardiac magnetic resonance imaging (**B**, **C**, **E**, **F**) showing a lesion (arrows) in the pericardial region, in close contact with the lateral wall of the left ventricle. In transthoracic echocardiography, a hyperechogenic mass is visualized in the apical three-chamber view (**A**) and the parasternal short-axis view focused on the left ventricle (**D**). Cardiac magnetic resonance imaging revealed a lesion with an adipose component in the three-chamber view (**B** and **C**) and the longitudinal axis of the ventricles (**E** and **F**). No late contrast enhancement was observed (**C**). In F, a hypointense signal is noted in the T2-weighted fat-saturation sequence, suggesting a lesion with a fatty component. LA: left atrium; RV: right ventricle; LV: left ventricle; Ao: aorta. Exams performed at the Heart Institute

Among the 69 evaluated patients, 42 (60.9%) had cardiac tumours, including 41 rhabdomyomas (Fig. 2) and one pericardial lipoma (Fig. 3). Table 1 presents the demographic data. Overall, patients with cardiac tumours were diagnosed at a markedly younger age, while the non-cardiac group showed a predominance of adult patients, without significant differences in sex or ethnicity distribution.

**Table 1:** Demographic variables of overall sample and patients with cardiac tumours

	Overall sample (*n* = 69)	Cardiac tumour sample (*n* = 42)	No cardiac tumour sample (*n* = 27)	*p* (< 0.05)
Female sex (*n*, %)	43 (62.3%)	21 (50%)	22 (81.5%)	0.11^(1)^
Age at diagnosis (median [Q1; Q3])	4.0 [0.0; 22.0]	0.5 [0.0; 6.2]	22.0 [13.5; 33.0]	**< 0.001** ^(2)^
Ethnicity				0.929^(3)^
White (n, %)	63 (91.3%)	39 (92.9%)	24 (88.9%)	
Mixed (n, %)	3 (4.3%)	2 (4.8%)	1 (3.7%)	
Asian (n,%)	2 (2.9%)	1 (2.4%)	1 (3.7%)	
Black (n,%)	0 (0%)	0 (0%)	0 (0%)	
Not reported (n,%)	1 (1.5%)	0 (0%)	1 (3.7%)	

Fisher’s exact test, (2) Wilcoxon-Mann-Whitney test, (3) Chi-square test

Subependymal nodules were the most frequent major criterion among patients with cardiac tumours (85.7%), whereas renal angiomyolipomas predominated in those without (92.6%), as shown in Table 2. Lymphangioleiomyomatosis (LAM), periungual fibromas, and renal/hepatic angiomyolipomas were significantly more prevalent in the group without cardiac tumours.

**Table 2:** Major diagnostic criteria in patients with and without cardiac tumours

Major criteria	Total sample (*n* = 69)	With cardiac tumours (*n* = 42)	Without cardiac tumours (*n* = 27)	*p* (< 0.05)
Hypomelanotic macules (n,%)	33 (47.8%)	20 (47.6%)	13 (48.2%)	> 0.999
Angiofibroma or fibrous cephalic plaque (n,%)	41 (59.4%)	21 (50%)	20 (74.1%)	0.078
Periungual fibromas (n, %)	22 (31.9%)	7 (16.7%)	15 (55.6%)	**0.001**
Shagreen patches (n, %)	12 (17.4%)	6 (14.3%)	6 (22.2%)	0.518
Retinal hamartomas (n, %)	7 (10.1%)	4 (9.5%)	3 (11.1%)	> 0.999
Cortical tubers (n, %)	49 (71%)	31 (73.8%)	31 (73.8%)	0.592
Radial migration lines (n, %)	24 (34.8%)	15 (35.7%)	9 (33.3%)	> 0.999
Subependymal nodules (n,%)	56 (81.2%)	36 (85.7%)	20 (74.1%)	0.344
SEGA (n,%)	12 (17.4%)	8 (19.1%)	4 (14.8%)	0.753
Cardiac rhabdomyoma (n,%)	41 (59.4%)	41 (97.6%)	0 (0%)	**< 0.001**
LAM (n,%)	19 (27.5%)	3 (7.1%)	16 (59.3%)	**< 0.001**
Angiomyolipomas (n,%)				
Renal	47 (68.1%)	22 (52.4%)	25 (92.6%)	0.002
Hepatic	11 (15.9%)	4 (9.5%)	7 (25.9%)	0.011
Soft tissue	1 (1.5%)	1 (2.4%)	0 (0%)	-
Pancreatic	1 (1.5%)	0 (0%)	1 (3.7%)	-

SEGA: subependymal giant cell astrocytoma; LAM: lymphangioleiomyomatosis. All comparisons were performed using Fisher’s exact test

Among the minor criteria (Table 3), sclerotic bone lesions were the most common in both groups. The presence of intraoral fibromas and sclerotic bone lesions was significantly more frequent in the group without cardiac tumours.

**Table 3:** Minor diagnostic criteria present in patients with and without cardiac tumours

Minor criteria	Total sample (*n* = 69)	With cardiac tumours (*n* = 42)	Without cardiac tumours (*n* = 27)	*p* (< 0.05)
“Confetti” skin lesions (n,%)	13 (18.8%)	6 (14.3%)	7 (25.3%)	0.344
Enamel pitting (n,%)	9 (13%)	3 (7.1%)	6 (22%)	0.139
Intraoral fibromas (n,%)	12 (17.4%)	4 (9.5%)	8 (29.3%)	**0.0496**
Retinal achromic patch (n,%)	0 (0%)	0 (0%)	0 (0%)	**-**
Multiple renal cysts (n,%)	17 (24.6%)	11 (26.2%)	6 (22.2%)	0.781
Sclerotic bone lesions (n, %)	31 (44.9%)	14 (33.3%)	17 (63%)	**0.025**

All comparisons were performed using Fisher’s exact test

On the other hand, seizures were more commonly reported in patients with cardiac tumours (90.5% versus 51.9%, *p* < 0.001) and were present in 75.4% of the overall sample. Delayed neuropsychomotor development (35.7% vs. 22.2%, *p* = 0.23) and autism spectrum disorder (11.9% vs. 14.8%, *p* = 0.73) did not significantly differ between the groups.

In summary, cardiac involvement was associated mainly with neurological manifestations, particularly seizures, whereas extracardiac structural manifestations — especially renal and pulmonary findings — predominated in patients without cardiac tumours.

According to sex-based comparative analysis, rhabdomyomas (76.9% vs. 48.8%, *p* = 0.02) were significantly more prevalent in males. Seizures (92.3% vs. 65.1%, *p* = 0.011) and delayed neuropsychomotor development (46.2% vs. 20.9%, *p* = 0.027) were also more common in males.

Genetic analysis was performed in only three patients (4.3% of the sample), identifying two mutations in the TSC2 gene and one negative result.

All-cause mortality data were confirmed for 89.9% of the sample. Overall mortality occurred in 8.06% of patients, with similar rates between groups (7.1% vs. 8.3%, *p* > 0.999). Malignant neoplasms were slightly more frequent in the group without cardiac tumours, but the difference was not statistically significant (11.1% vs. 4.8%, *p* = 0.37). The use of mTOR inhibitors was more frequent in the group without cardiac tumours (44.4% vs. 14.3%, *p* = 0.005) and was significantly higher among females compared to males (37.2% vs. 7.69%, *p* < 0.01).

Most patients with cardiac tumours were asymptomatic (73.8% and 85.7% across assessments). Dyspnea was the most reported symptom (Table 4).

**Table 4:** Evolutionary data of patients with cardiac involvement (*n* = 42)

	First Assessment	Last Assessment	*p* (< 0,05)
Age, years (median [Q1; Q3])	3.5 [1.0; 15.8]	12.5 [5.0; 24.5]	
< 18 years (n, %)	33 (78.6%)	23 (54.8%)	**< 0.001** ^**2**^
*≥* 18 years (n, %)	9 (21.4%)	19 (45.2%)	**< 0.001** ^**3**^
Body surface area, m² (mean, SD)			
Paediatric population (Haycock)	0.71 ± 0.51	1.19 ± 0.58	**< 0.001** ^1^
Adult population (DuBois)	1.71 ± 0.23	1.74 ± 0.19	0.139^1^
*Clinical manifestations*			
Atypical chest pain (n, %)	4 (9.5%)	1 (2.4%)	0.180^3^
Dyspnea (n, %)	5 (11.9%)	3 (7.1%)	0.414^3^
Palpitations (n, %)	2 (4.8%)	2 (4.8%)	> 0.999^3^
*Echocardiographic data*			
LA (mean, SD)			
< 18 years (Z-score)	0.56 ± 0.93	0.52 ± 1.48	0.904^1^
*≥* 18 years (mm/m²)	19.18 ± 2.00	18.40 ± 2.04	0.734^2^
Septum (mean, SD)			
< 18 years (Z-score)	1.04 ± 1.31	0.52 ± 1.24	**0.049** ^1^
*≥* 18 years (mm/m²)	4.97 ± 0.44	4.61 ± 0.47	0.080^1^
Posterior wall (mean, SD)			
< 18 years (Z-score)	1.24 ± 1.54	0.53 ± 0.98	**0.017** ^1^
*≥* 18 years (mm/m²)	4.97 ± 0.47	4.61 ± 0.47	0.077^1^
LVEDD (mean, SD)			
< 18 years (Z-score)	0.01 ± 1.25	-0.36 ± 1.68	0.259^1^
*≥* 18 years (mm/m²)	25.79 ± 2.92	24.45 ± 2.73	0.075^1^
RWT (mean, SD)	0.37 ± 0.07	0.35 ± 0.07	0.117^1^
Aortic root (mean, SD)			
< 18 years (Z-score)	0.52 ± 1.57	0.25 ± 1.12	0.285^1^
*≥* 18 years (mm/m²)	15.24 ± 1.74	16.93 ± 0.93	**0.020** ^1^
LVEF Teichholz, % (mean, SD)	67.88 ± 7.37	67.03 ± 6.06	0.364^1^
RV dilatation (n, %)	3 (7.1%)	1 (2.4%)	0.157^3^
Pericardial effusion (n, %)	1 (2.4%)	1 (2.4%)	> 0.999^3^
Haemodynamic repercussion (n, %)	2 (4.8%)	0 (0%)	0.157^3^

LA: Left atrium anteroposterior diameter; LVEDD: Left ventricular end-diastolic diameter; RWT: Relative wall thickness; LVEF: Left ventricular ejection fraction; RV: Right ventricle. (1) Paired t-test, (2) Paired Wilcoxon test, (3) Asymptotic McNemar test

Among patients with cardiac tumours, supraventricular and ventricular tachycardia were observed in 16.7% and 7.2% of cases, respectively, while Wolff-Parkinson-White syndrome was present in 9.5%. Overall, arrhythmias occurred in 21.4% of patients at some point during follow-up.

Regarding medication use, 23.8% of patients used beta-blockers, 14.3% used renin-angiotensin-aldosterone system inhibitors, 11.9% used diuretics, and 7.1% used amiodarone.

The median follow-up period between the first and last TTE performed was 6 years [2.2; 9.8]. Throughout the follow-up period, there was no clinically significant change in the echocardiographic data analyzed (Table 4). Only one patient (2.4%) had an LVEF < 50%. Overall, echocardiographic parameters remained stable over time, indicating preserved ventricular function and minimal structural progression despite tumour persistence.

Rhabdomyomas most commonly involved the left ventricle or both ventricles (43.9% each). Among those with multiple tumours, biventricular involvement was most common (58.1%, Table S1).

Holter monitoring was performed in 20 patients, revealing sinus rhythm in 60%, sinus rhythm with atrial ectopy in 35%, and sinus arrhythmia in 5%. Simple and complex arrhythmias were observed in 25% and 5% of patients, respectively.

CMR data were available for 15 patients with cardiac tumours, with 20% showing late gadolinium enhancement (LGE). In 13.3% of these cases, tumours were detected exclusively by CMR and were not identified on the initial TTE.

Incomplete regression was the most common tumour outcome (76.2%), with complete regression occurring in 16.7% of patients (Figure S1). Only one patient (2.4%) experienced tumour growth, and another had a stable tumour size. Surgical intervention was required in one case, which was followed by death due to refractory arrhythmia (2.4%). No patient underwent heart transplantation or received an implantable cardioverter-defibrillator, whereas three patients (7.14%) underwent catheter ablation.

The patient with pericardial lipoma maintained tumour size stability, but required ablation for epicardial ventricular tachycardia, remaining asymptomatic afterward.

An association analysis (Table 5) revealed a higher prevalence of complex arrhythmia in patients with progression events (tumour growth, surgery, or death; *p* = 0.051). However, it was not possible to correlate complex arrhythmias on Holter with CMR-LGE due to the small number of available exams in the sample. Ordinal logistic regression revealed no significant predictors of clinical progression (Table S2).

**Table 5:** Association of outcomes with selected sociodemographic and clinical variables

	Complete regression (*n* = 7)	Incomplete regression (*n* = 32)	Mass maintenance, increase, or surgical treatment (*n* = 3)	*p* (< 0.05)
Age, years (median [Q1; Q3])	1.0 [0.5; 1.5]	0.0 [0.0; 9.2]	0.0 [0.0; 7.5]	0.952
Female sex (n, %)	4 (57.1%)	16 (50.0%)	1 (33.3%)	0.788
Multiple tumours	5 (71.4%)	24 (75.0%)	2 (66.7%)	0.940
LVEF < 50% at presentation	0 (0.0%)	1 (3.1%)	0 (0.0%)	-
Late gadolinium enhancement on CMR	1 (50%)	1 (9.1%)	1 (50%)	0.216
Complex arrhythmias on Holter	0 (0%)	0 (0%)	1 (33.3%)	0.051
Use of mTOR inhibitor	1 (14.3%)	4 (12.5%)	1 (33.3%)	0.615

LVEF: left ventricular ejection fraction; CMR: cardiac magnetic resonance; mTOR: mammalian target of rapamycin

## Discussion

This study presents a comprehensive analysis of the clinical features, systemic involvement, and disease progression in Brazilian patients with TSC-associated rhabdomyomas.

As an autosomal dominant condition, TSC typically shows no sex difference; however, our cohort had a female predominance (62.3%), especially among those without cardiac tumours (81.5%). This finding is possibly attributed to the high prevalence of patients with LAM (59.3%) in this group, considering that our centre also specializes in pulmonology.

Interestingly, rhabdomyomas were less common in females, a finding not previously reported [10, 11]. This may be associated with higher mTOR inhibitor use among females (37.2% versus 7.7%). In 2021, Chen et al. described increased rhabdomyoma resolution with the use of mTOR inhibitors, particularly in women and younger patients [28]. Thus, female patients might have experienced faster tumour regression, making them undetectable via echocardiography at the initial follow-up.

However, in our study, female sex and mTOR use did not correlate with higher complete regression. This may reflect more detailed imaging in previously diagnosed patients, including more frequent CMR use, which improves detection of small lesions.

Another relevant finding was the significantly younger age at diagnosis in the group with cardiac involvement. This finding, previously reported in other centres [1], is likely due to the earlier diagnosis of rhabdomyomas, often made during the prenatal period through foetal echocardiography. Furthermore, rhabdomyomas have been described as the most frequent manifestation in children up to 36 months of age and are present in 59% of cases [29, 30].

The prevalence of rhabdomyomas in our institution was 59.4%, which is higher than the reported range in the literature [10, 11]. This difference is likely associated with our status as a national reference centre in paediatric and adult cardiology, which may have led to underrepresentation of patients without cardiac tumours.

A rare case of pericardial lipoma, unreported in previous literature, was documented in a male patient with ventricular tachycardia and a stable tumour size.

Systemic manifestations were consistent with other reports, except for a lower occurrence of cutaneous, oral, retinal, and sclerotic bone lesions [31]. These data may be underestimated, as they were based on medical records filled out by cardiologists and pulmonologists.

Seizures were significantly more prevalent in patients with cardiac tumours (90.5% vs. 51.9%, *p* = 0.0003). In 2016, Jeong and Wong reported that rhabdomyomas were the only systemic manifestation that remained significantly associated with epilepsy in multivariate analysis [32], consistent with our findings. However, there were no differences in identifiable anatomical lesions on the neuroimaging exams. This suggests that the pathophysiology of seizures remains unclear and warrants further investigation.

Recently, in 2022, Samuel YL et al. reported a greater occurrence of cardiac rhabdomyomas, renal angiomyolipomas, and facial angiofibromas in Chinese patients with TSC2 mutations, suggesting a possible association between cardiac tumours and more severe systemic involvement [33]. This association was not found in our study, as systemic manifestations such as renal angiomyolipomas, LAM, sclerotic bone lesions, and intraoral and periungual fibromas were more common in the group without cardiac involvement. Genetic analysis would certainly provide relevant insights into the genotype-phenotype correlation in our population. However, only 4.3% of patients underwent genetic testing due to limited availability in the public healthcare system, restricting interpretation.

All-cause mortality was slightly higher in the non-cardiac group (8.3% versus 7.1%), without statistical significance. A 2017 study reported renal disease as the leading cause of mortality in TSC [34]. Although specific causes of death were unavailable, the higher prevalence of renal angiomyolipomas in the non-cardiac group may partially explain this finding.

In our sample, cardiac tumours were predominantly multiple in 75.6% of cases, a proportion slightly lower than the 90% reported in other studies [9]. This may be associated with the predominant use of transthoracic echocardiography in our study.

Most patients with cardiac rhabdomyomas remained asymptomatic during follow-up (73.8% at the first and 85.71% at the last evaluation). In 2006, Jóźwiak et al. evaluated 74 patients with TSC-associated rhabdomyomas and reported that 61% of tumours had no clinical manifestations, with only one death directly related to cardiac involvement (1.4%) and a 5.4% incidence of heart failure [11]. In 1996, Bosi et al. reported only one case of heart failure (3.3%) in a series of 30 TSC-associated rhabdomyomas, with no deaths directly related to cardiac involvement and no tumour-related surgical indications [35]. Our data also revealed a rare occurrence of these outcomes, with only one heart failure case, one death, and one surgical intervention in the same patient (2.4%).

Jóźwiak et al. reported a 23% arrhythmia rate [11], whereas Bosi et al. found a 26.7% rate [35]. Our study showed similar results (21.4% arrhythmias, with 16.7% supraventricular tachycardias and 7.2% ventricular tachycardias). Nir et al. described Wolff-Parkinson-White syndrome in 9% of patients with rhabdomyomas, also consistent with our findings (9.5%) [36].

To date, no longitudinal studies have evaluated the progression of TSC-associated rhabdomyomas in the Brazilian population. Most published data on cardiac tumours in patients with tuberous sclerosis complex derive from high-income settings, particularly European cohorts and international registries largely composed of centres in developed countries. Evidence from middle-income regions is limited and typically restricted to small single-centre series. This is relevant, as variations in access to genetic testing, imaging resources and structured follow-up may influence both diagnostic timing and the observed natural history of the disease.

Our complete regression rate (16.7%) was lower than that in recent reports [10, 37], with incomplete regression being the most common clinical outcome (76.2%). This difference is likely due to advancements in imaging techniques, which allow for higher sensitivity in detecting smaller tumours. Furthermore, adult patients were also included, whereas complete regression is classically described in early childhood.

Finally, the median follow-up period was six years, primarily determined by the availability of electronic medical records. While this timeframe likely suffices for characterizing early tumour evolution, longer follow-up studies could be useful in assessing possible tumour growth or new tumour development during puberty.

### Methodological considerations and limitations

The availability of data was conditioned by electronic records made by different professionals over 27 years. During this period, there was an improvement in imaging techniques and a progressive migration to more computerized systems. Undoubtedly, the application of standardized questionnaires would provide more accurate and uniform information on systemic and cardiological manifestations in patients with TSC.

Tumour evolution was primarily assessed through serial transthoracic echocardiography performed by different operators (adult and paediatric teams). Additionally, these patients were evaluated using the Z-score calculation for linear measurements throughout the follow-up, even if they were over 18 years old at the last examination. Although this methodology has not been validated, it has been used to facilitate the evolutionary comparison of variables, which may have impacted the results.

Although CMR is the gold standard for rhabdomyoma diagnosis, its routine use was limited in this paediatric population due to sedation requirements. Therefore, inferences related to the presence of late enhancement in tumour prognosis are limited by the small number of examinations performed.

Histologically, cardiac rhabdomyomas are benign hamartomatous lesions composed of enlarged, vacuolated cells with sparse cytoplasm that resemble altered myocytes, with high glycogen content. “Spider cells” are typical and are characterized by a centrally located nucleus with radial extensions to the cell periphery [38]. Although histological confirmation was not performed in this cohort — as diagnosis was based on typical imaging findings — these well-established pathological features support the biological profile of rhabdomyomas and their distinction from other paediatric cardiac masses. Future studies integrating imaging and pathological correlation may provide further insight into tumour behaviour and progression.

Another important limitation was the low availability of genetic testing, which prevents the establishment of a genotype-phenotype correlation in the studied population.

Additionally, this was a single-centre retrospective study, which may not represent the Brazilian population in general. Given the still incompletely understood pathophysiology of TSC, population-specific studies may reveal interactions between genetic and environmental, nutritional, racial, and epidemiological factors. However, the rarity of TSC, limited resources, and diagnostic challenges hinder multicentre research on this topic in Brazil.

In this study, a low mortality rate of only 2.4% was found. The Brazilian foetal tumour cohort published in 2024 by Camargo et al. reported a higher mortality rate of 17.4% [20]. However, most deaths (72.7%) occurred in the foetal or early neonatal period. Since our centre does not have its own maternity service and does not provide prenatal follow-up, most patients with cardiac tumours begin follow-up at an outpatient level after the initial peak of reported deaths, which explains the discrepancy in rates reported.

Although mortality significantly decreases after the neonatal period, most patients retain identifiable tumours in sequential examinations, representing potential arrhythmogenic substrates. In agreement with this, a significant rate of cardiac arrhythmias was found, reinforcing the importance of specialized longitudinal follow-up in cardiology.

In terms of treatment, the use of mTOR inhibitors was not associated with improved clinical outcomes. However, this finding should be interpreted with caution, considering the low occurrence of heart failure, death, and the need for surgical intervention in the sample. Randomized studies may clarify the potential benefits of these medications for cardiac rhabdomyomas, which are currently reserved for symptomatic cases or large-volume masses.

## Conclusions

In this cohort, incomplete remission was the most frequent outcome of cardiac tumours evaluated through serial echocardiograms. Cardiac tumours are associated with a considerable occurrence of arrhythmias during follow-up, exceeding 20%, despite low mortality rates and limited surgical treatment.

Cardiac rhabdomyomas were associated with a higher occurrence of seizures, while renal angiomyolipomas, LAM, sclerotic bone lesions, intraoral and periungual fibromas were more frequent in patients without cardiac involvement.

No factors were found to be associated with the progression of cardiac tumours among the variables analyzed in the sample (use of mTOR inhibitors, presence of late gadolinium enhancement on CMR, LVEF < 50% at presentation, female sex, age, and multiple tumours). Further studies with larger samples and longer follow-up periods are needed to clarify the impact of these variables.

## Supplementary Information

Below is the link to the electronic supplementary material.

Supplementary Material 1

## Data Availability

The datasets generated and/or analysed during the current study are available from the corresponding author on reasonable request.

## Abbreviations

CMR: Cardiac magnetic resonance imaging
LAM: Lymphangioleiomyomatosis
LGE: late gadolinium enhancement
LVEF: left ventricular ejection fraction
mTOR: Mammalian target of rapamycin
TSC: Tuberous sclerosis complex
TTE: Transthoracic echocardiography
