# Supplementary material for: Original investigation: evolution of long-term cardiac tumours in patients with tuberous sclerosis
Source: Orphanet J Rare Dis. 2026 May 7;21:184. doi: 10.1186/s13023-026-04302-9 (PMC13154433; doi:10.1186/s13023-026-04302-9)

**Supplemental Materials**

Table S1 ‒ Tumor location in patients with cardiac rhabdomyomas

| **Location** | **Single rhabdomyomas**  **(n = 10)** | **Multiple rhabdomyomas**  **(n = 31)** | **Total**  **(n = 41)** | **p (< 0.05)** |
| --- | --- | --- | --- | --- |
| Isolated RV involvement | 2 (20%) | 0 (0%) | 2 (4.9%) | 0.055 |
| Isolated LV involvement | 7 (70%) | 11 (35.5%) | 18 (43.9%) | 0.075 |
| Isolated RA involvement | 1 (10%) | 0 (0%) | 1 (2.4%) | 0.244 |
| Biventricular involvement | 0 (0%) | 18 (58.1%) | 18 (43.9%) | **0.002** |
| Biventricular and RA involvement | 0 (0%) | 1 (3.2%) | 1 (2.4%) | - |
| LV and RA involvement | 0 (0%) | 1 (3.23%) | 1 (2.4%) | **-** |

RV: right ventricle; LV: left ventricle; RA: right atrium. All comparisons performed using Fisher’s exact test.

Table S2: Ordinal logistic regression model results for outcomes among patients with cardiac tumors

| **Variable** | **Odds Ratio [IC95%]** | **p (< 0.05)** |
| --- | --- | --- |
| Age (years)  Female sex | 1.06 [0.97 ;1.17]  0.37 [0.07 ;1.83] | 0.215  0.230 |
| Use of mTOR inhibitor | 1.25 [0.12 ;14.78] | 0.853 |
| Multiple tumors | 0.91 [0.17 ;4.61] | 0.912 |

mTOR: mammalian target of rapamycin protein

Figure S1 - Clinical course of patients with cardiac involvement
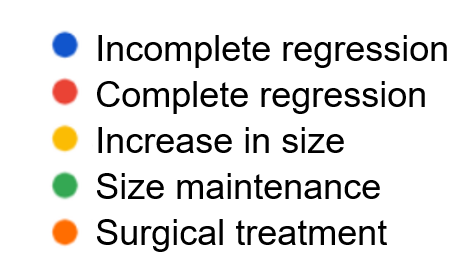


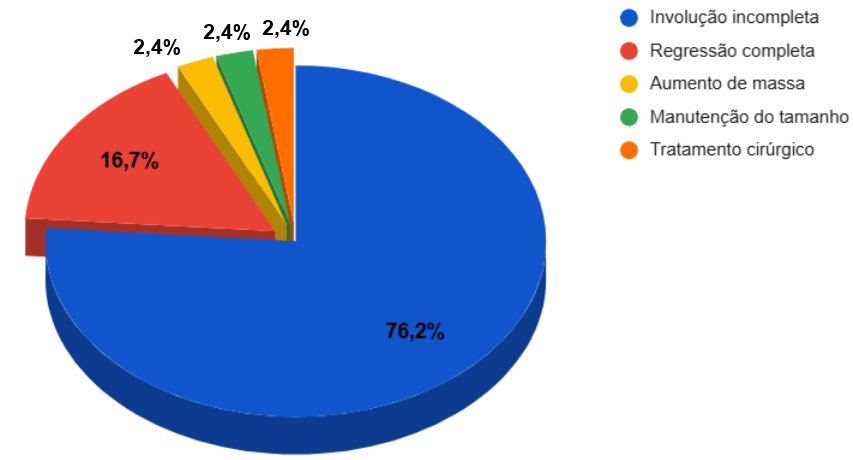

Supplement: Supplementary file 1 — Supplementary Material 1 [file 13023_2026_4302_MOESM1_ESM.docx]
